# Sellar Melanoma With Lung Metastasis: A Case Report

**DOI:** 10.7759/cureus.56469

**Published:** 2024-03-19

**Authors:** Lukas Goertz, Christoph Kabbasch, Marco Timmer

**Affiliations:** 1 Department of Radiology, University Hospital Cologne, Cologne, DEU; 2 Department of General Neurosurgery, Center for Neurosurgery, University Hospital Cologne, Cologne, DEU

**Keywords:** whole-brain radiation, brain and lung metastasis, transsphenoidal neurosurgery, sella, melanoma

## Abstract

We report the case of an 81-year-old patient with no pre-existing medical conditions who presented with a one-week history of progressive horizontal diplopia. Contrast-enhanced brain magnetic resonance imaging showed a heterogeneous sellar mass with the infiltration of the cavernous sinus and sella. Hormone levels were within normal limits. Considering an endocrine inactive pituitary adenoma, the patient underwent transsphenoidal resection. After surgery, the preoperative symptoms completely resolved. Histopathologic examination of the tumor specimen revealed melanoma. Since the patient had no history of cancer, an extensive staging workup was performed, which revealed multiple lung metastases. However, no primary tumor was found. We recommended adjuvant brain irradiation and chemo- and immunotherapy, but the patient refused further oncological treatment and died five months after surgery. Reported cases of sellar melanoma are rare, and the combination of sellar melanoma and lung metastasis without a cutaneous primary is unique. Although rare, malignant lesions of the sella must be considered in the differential diagnosis, especially in cases with rapid onset of symptoms.

## Introduction

Malignant melanoma, a type of skin cancer that arises from pigment-producing cells known as melanocytes, is known for its aggressive nature and propensity to metastasize. While it predominantly spreads to regional lymph nodes and distant organs such as the lungs, liver, and brain, sella involvement is extremely rare and rarely documented in the medical literature [1,2]. The rarity of such cases may be attributed to the unique anatomical and vascular characteristics of the pituitary gland, as well as the distinctive molecular characteristics of melanoma cells. The literature suggests that the majority of melanoma cases with sellar metastases occur in patients with a history of cutaneous melanoma. However, the occurrence of primary melanoma arising from the sella is even more unusual. We report a rare case of sellar melanoma with concomitant lung metastases in a patient with no evidence of cutaneous melanoma.

## Case presentation

An 81-year-old female presented to our emergency department with a one-week history of progressive horizontal diplopia and convergent strabismus of the left eye. The patient was alert, fully oriented, and in good general health. She denied headaches and signs of inflammation. The neurological examination revealed left abducens palsy and known right visual loss. The left abducens nerve was intact. The neurological examination also revealed visual impairment in the left eye and bitemporal hemianopsia. The other cranial nerves were intact, and no other neurological deficits were found. The patient did not report any history of cancer, and no relevant pre-existing diseases were known. Clinical examination revealed no other abnormalities. Serum chemistry and complete blood count were within normal limits.

**Figure 1:**
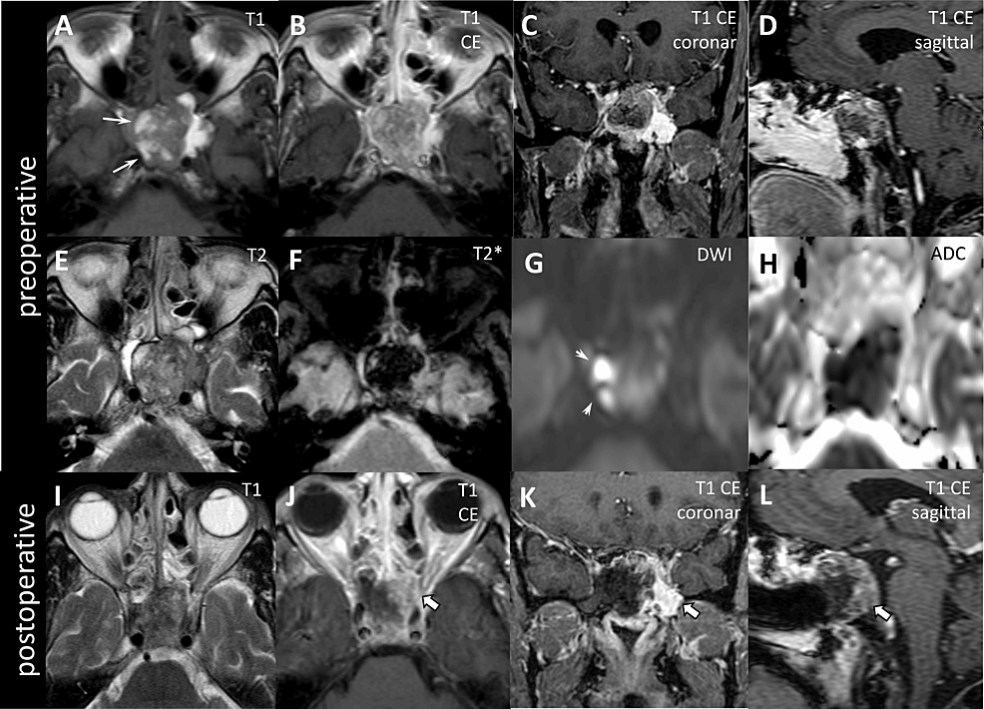
Pre- and postoperative imaging. The figure shows preoperative (A-H) and postoperative (I-L) MR images in axial reconstructions unless otherwise indicated. T1-weighted MRI shows a sellar mass with focal hyperintense spots (arrows) indicating melanin content (A). CE T1 images show inhomogeneous enhancement and infiltration of surrounding structures (B-D). The mass is inhomogeneous on T2 images (E) and has a low signal on susceptibility-weighted images (T2*, F). The melanin-containing areas are diffusion-impaired, as shown by a high signal on DWI (G) and low ADC values (H). Postoperative T1 (I) and T1 CE show residual tumors at the left sella and the left cavernous sinus (block arrows, J-L). MRI: magnetic resonance imaging; CE: contrast-enhanced; DWI: diffusion-weighted images; ADC: apparent diffusion coefficient

Emergency head computed tomography (CT) scan with bone windows showed a solitary large hyperdense sellar mass lesion extending to the sphenoid sinus with partial bony destruction of the clivus. Contrast-enhanced brain magnetic resonance imaging (MRI) was subsequently performed and showed a multinodular sellar mass with supra- and infrasellar proportions and infiltration into the left cavernous sinus (Figure 1). 

The lesion was isointense on T1-weighted images with hyperintense spots. The lesion was heterogeneous on T2-weighted sequences. Contrast-enhanced MRI showed inhomogeneous enhancement within the mass. The lesion showed invasion of the adjacent paranasal and cavernous sinuses with bilateral internal carotid artery involvement and compression of the left optic nerve. There was no evidence of other intracranial lesions. Based on the imaging findings, differential diagnoses of pituitary macroadenoma, sellar meningioma, and sellar metastasis of unknown origin were considered.

As a result, we performed a hormone test, which revealed a slightly elevated prolactin level (33.2 µg/L; reference range: 6.0-29.9 µg/L), which was interpreted as a stalk effect. Other hormone levels were within normal limits as shown in Table 1.

**Table 1:** Hormone levels of the patient at baseline. TSH: thyroid-stimulating hormone; LH: luteinizing hormone; FSH: follicle-stimulating hormone; GH: growth hormone; mU: milliunit; L: liter; ng: nanogram; U: unit; µg: microgram

Hormone	Patient level	Reference level
TSH	2.23 mU/L	0.27-4.20 mU/L
Triiodothyronine (free T3)	2.4 ng/L	2.0-4.4 ng/L
Thyroxine (free T4)	10.8 ng/L	9-17 ng/L
LH	15.4 U/L	7.7-58.5 U/L
FSH	35.2 U/L	25.8-134.8 U/L
Prolactin	33.2 µg/L	6.0-29.9 µg/L
GH	0.6 µg/L	0-8 µg/L

Assuming an endocrine inactive macroadenoma of the pituitary gland, surgical resection of the tumor via a transsphenoidal approach was planned. Due to the rapid onset of symptoms and the size of the tumor, surgery was performed within three days of the patient's initial presentation to our hospital via an endoscopic endonasal transsphenoidal approach. A soft, partly papillomatous, partly necrotic mass with hyperpigmented parts was seen, which was removed partially from the right to the left side of the sphenoid sinus. Due to cavernous sinus infiltration and encasement of both internal carotid arteries, gross tumor removal could not be achieved, but a small portion of the tumor in the left cavernous sinus had to be left. Postoperatively, the patient received prophylactic penicillin antibiosis and hydrocortisone substitution. The initial symptoms completely resolved within a few days, and the patient was free of complaints. Postoperative blood tests showed no evidence of hypopituitarism or diabetes insipidus.

**Figure 2:**
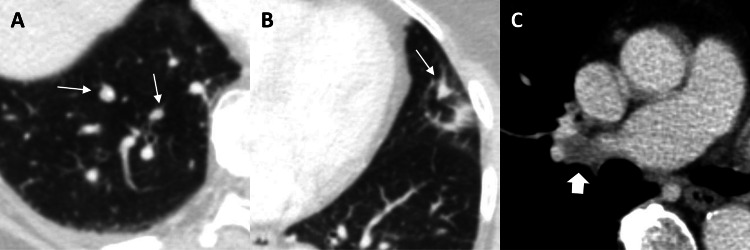
Staging CT. Lung CT shows multiple pulmonary lesions, exemplary in the right (A) and left (B) lower lobes (arrows). There was also a malignant suspicious lymph node in the right hilar region (C, block arrow), which together indicated metastasis. CT: computed tomography

Histopathological examination of the tumor sample revealed a malignant tumor with melanotic pigmentation, consistent with malignant melanoma. Unfortunately, the immunohistopathological characterization of the tumor has not been adequately documented and archived. As there was no history of melanoma, an extensive dermatologic and ophthalmologic workup was performed, which revealed no evidence of cutaneous or uveal primary melanoma. A dermal lesion was resected and histopathology revealed it to be a fibroma. CT staging of the chest and abdomen showed multiple pulmonary lesions in all lobes with a maximum size of 8 mm and a malignant suspicious hilar lymph node, indicating metastasis (Figure 2). However, they were considered too small for biopsy. No pathologically enlarged lymph nodes or other metastases were found. To further clarify the origin of the tumor, an esophagogastroduodenoscopy and coloscopy were performed to rule out gastrointestinal melanoma. No evidence of a primary tumor or additional metastases was found. Because of the metastatic disease, we recommended adjuvant brain irradiation and dermato-oncologic treatment consisting of chemo- and immunotherapy. As the patient refused further oncologic treatment, she was discharged from the hospital in good general condition. The patient did not return for her scheduled three-month follow-up and died five months after her initial presentation to our hospital, presumably due to tumor progression; however, follow-up imaging was not performed.

## Discussion

Clinically apparent pituitary metastases are rare, accounting for only 1% of sellar lesions. Among these, brain and lung carcinomas have been shown to be the most common primary sites [3]. Cerebral metastasis of malignant melanoma is a common feature and a sign of advanced cancer stage. Sellar melanoma metastases have been described as incidental findings in autopsy series, associated with widespread systemic disease and multiple intra- and extracranial metastases. In contrast, symptomatic solitary sellar melanoma is very rare, and only a few cases have been reported.

In the literature, pituitary melanoma is divided into primary and metastatic melanoma. Primary sellar melanoma was considered when there was no evidence of primary cutaneous melanoma and no known history of skin cancer. A plausible explanation for the development of primary melanoma is the malignant degeneration of melanin-bearing cells in the surrounding leptomeninges and subsequent migration into the pituitary [4]. In contrast, sellar metastasis is considered a metastasis if there is a known history of melanoma [2]. Possible routes of metastasis to the sella include hematogenous spread to adjacent tissues such as the posterior lobe, pituitary stalk, or clivus with subsequent spread to the sella.

In this report, we present an unusual case of solitary sellar melanoma with concomitant pulmonary metastasis. Therefore, both primary sellar melanoma with subsequent pulmonary metastasis and metastatic melanoma need to be considered. As we could not present the results of an immunohistochemical study, the differentiation between primary sellar melanoma and sellar metastasis cannot be made. Indeed, spontaneous regression of cutaneous melanoma has been described in several studies [5]. In our case, a primary skin tumor could have formed metastatic lesions and then spontaneously regressed or decreased in size. In fact, the rapid onset and progression of symptoms and concomitant lung metastases make sellar metastasis more likely. 

Differentiation between benign pituitary tumor and sellar metastasis is important because it is associated with different treatment outcomes. While only histopathologic analysis of the tumor specimen can definitively determine the type of tumor, the patient's history, clinical examination, and imaging studies can provide valuable clues to the type of tumor. On MRI, differentiation between pituitary adenoma and melanoma primary/metastasis can sometimes be difficult because both entities are similar in shape, cavernous sinus invasion, and intratumoral hemorrhage. However, melanoma has a characteristic pattern on MRI due to its melanin content: It has high intensity on T1-weighted MR images and appears hypointense on T2-weighted images. In contrast, a pituitary adenoma tends to appear hyperintense on T2-weighted images and hypointense on T1-weighted images and shows more homogeneous contrast enhancement than a metastasis.

If the patient is in good general condition, transsphenoidal surgery is the treatment of choice to treat sellar mass lesions, relieve symptoms, and obtain tumor tissue for histopathologic analysis. If the pathohistological examination reveals a sellar metastasis, a comprehensive staging workup including dermatologic, ophthalmologic, gastrointestinal, and radiologic staging is necessary to assess tumor progression and identify a primary tumor. This is a prerequisite for establishing an individualized treatment regimen including radiation and chemotherapy, consistent with current guidelines for the World Health Organization (WHO) grade IV melanoma. However, the occurrence of sellar or distant melanoma metastases is an indication of progressive tumor stage and poor overall survival, requiring adjuvant therapy. For local brain metastases, adjuvant therapy options include whole-brain irradiation, stereotactic radiosurgery, surgical resection, and systemic chemotherapy. In this case, the patient refused adjuvant therapy, resulting in death within five months of surgery.

## Conclusions

The presented cases show that sellar melanoma is a rare differential diagnosis of sellar lesions and may mimic hormonally inactive pituitary adenoma. The simultaneous detection of sellar melanoma and concomitant lung metastases is unique in the available literature, as it complicates the differentiation between primary sellar melanoma and sellar metastasis. This requires extensive staging to find a potential primary tumor. Rapid onset of symptoms and concomitant lung metastasis indicate metastatic melanoma in the presented case. Treatment consists of surgical resection and adjuvant oncologic therapy, following the guidelines for metastatic melanoma.
